# Non-Invasive Detection of Tumors by Volatile Organic Compounds in Urine

**DOI:** 10.3390/biomedicines13010109

**Published:** 2025-01-06

**Authors:** Tomoaki Hara, Sikun Meng, Yasuko Arao, Yoshiko Saito, Kana Inoue, Aya Hasan Alshammari, Hideyuki Hatakeyama, Eric di Luccio, Andrea Vecchione, Takaaki Hirotsu, Hideshi Ishii

**Affiliations:** 1 Department of Medical Data Science, Center of Medical Innovation and Translational Research, Osaka University Graduate School of Medicine, Suita, Yamadaoka 2-2, Osaka 565-0871, Japan; 2 Hirotsu Bio Science Inc., Chiyoda-Ku, Tokyo 102-0094, Japan; 3 Department of Clinical and Molecular Medicine, University of Rome “Sapienza”, Santo Andrea Hospital, Via di Grottarossa, 1035-00189 Rome, Italy

**Keywords:** cancer, biomarkers, diagnostics, volatile organic compounds, olfactory receptors

## Abstract

Cancer is one of the major causes of death, and as it becomes more malignant, it becomes an intractable disease that is difficult to cure completely. Therefore, early detection is important to increase the survival rate. For this reason, testing with blood biomarkers is currently common. However, in order to accurately diagnose early-stage cancer, new biomarkers and diagnostic methods that enable highly accurate diagnosis are needed. This review summarizes recent studies on cancer biomarker detection. In particular, we focus on the analysis of volatile organic compounds (VOCs) in urine and the development of diagnostic methods using olfactory receptors in living organisms. Urinary samples from cancer patients contain a wide variety of VOCs, and the identification of cancer specific compounds is underway. It has also been found that the olfactory sense of organisms can distinguish cancer-specific odors, which may be applicable to cancer diagnosis. We explore the possibility of novel cancer biomarker candidates and novel diagnostic methods.

## 1. Introduction

Early cancer treatment has a significant impact on subsequent survival [1,2,3]. Therefore, accurate diagnosis of early-stage cancer is important. Currently, CEA, CA19-9, and CA15-3 are mainly used as blood biomarkers for cancer diagnosis [4]. CEA, a glycoprotein, is a type of cell adhesion molecule that is normally expressed in intestinal, pancreatic, and liver cells during fetal life, but its expression increases in colon, stomach, lung, and breast cancers [5]. CA19-9 is a carbohydrate antigen called Sialyl Lewis(a), which is present in glycoproteins and glycolipids of Lewis-positive individuals and is expressed in small amounts in normal pancreatic, bile duct, stomach, and intestinal cells, but its production is increased in cancer cells [6]. CA15-3, part of a glycoprotein called mucin 1, is present in many epithelial cells, including mammary gland cells, and is overexpressed in breast cancer cells [7]. While these are useful cancer markers, they are not sufficient for the accurate diagnosis of early-stage cancer [8,9,10]. Biopsies are also used for cancer diagnosis, but it places a heavy burden on the patient [11]. Therefore, a search for novel cancer biomarkers in urine is being conducted to diagnose cancer in a non-invasive manner [12]. Urine contains numerous volatile organic compounds (VOCs), some of which have been found to have cancer-specific changes [13]. It has also been reported that organisms can detect cancer-specific odors [14]. Dogs and nematodes, in particular, are being studied for application to cancer diagnosis, and further improvement of the system is expected [15,16]. Development of cancer diagnosis methods using diagnostic devices based on inorganic substances is also underway [17]. This study summarizes the latest research on the search for cancer markers and the development of diagnostic methods.

## 2. Volatile Organic Compounds (VOCs) in Urine

Urine contains a variety of VOCs (Table 1). Studies are underway to apply them to cancer diagnosis. Analysis of VOCs in urine is possible with a mass spectrometer. Urine samples from 115 individuals were analyzed by headspace using gas chromatography coupled to ion mobility spectrometry (GC-IMS) and ketones (acetone, 2-butanone, 2-pentanone, 4-heptanone, and 2-hexanone), aldehydes (propanal, pentanal, hexanal, 3-methylbutanal and heptanal), sulfur compounds (dimethylsulphide and diallylsulphide), alcohols (ethanol, propanol, pentanol, 2-methyl-1 propanol, 2-methyl-1-butanol, and 2-hexanol), esters (butylacetate, pentylacetate, and ethylacetate), aromatic hydrocarbon (toluene), and terpene (α-pinene) were detected [18]. Urinary VOCs from various cancer patients have been analyzed using these methods.

### 2.1. VOCs of Lung Cancer

VOCs in the urine of lung cancer patients and healthy controls were examined by high-resolution mass spectrometry (HRMS) and nine VOCs (N-acetyl-S-methyl-L-cysteine, 3/4-methylhippuric acid, 2-methyl hippuric acid, N-acetyl-S-(N-methyl carbamoyl)-L-cysteine, N-acetyl-S-(2-carbamoylethyl)-L-cysteine, N-acetyl-S-(2-carboxyethyl)-L-cysteine, N-acetyl-S-(3-hydrox ypropyl-1-methyl)-L-cysteine, N-acetyl-S-(3-carboxy-2-propyl)-L cysteine, N-acetyl-S-(2-carbamoylethyl)-L-cysteine and N-acetyl-S (3,4-dihydroxybutyl)-L-cysteine) derived from aldehydes, alkenes, amides, and aromatics were detected in over 90% of both samples. Analysis of metabolites related to the release of VOCs revealed the presence of amino acid metabolism, steroid hormone biosynthesis and metabolism. The analysis of metabolites related to VOC release showed that amino acid metabolism, steroid hormone biosynthesis and metabolism, and fatty acid β-oxidation were related to VOC exposure, and 16 metabolites (nona-4,6-dienoylcarnitine, thymol sulfate, tetrahydrocortisone, 6-methylnonanoylcarnitine, 4-oxodecanoylcarnitine, 21-hydroxy-5beta-pregnane-3,11,20-trione, (2E,6E,8E)-decatrienoylcarnitine, 7-methyladenine, o-methylmalonyl-L-carnitine, 7-methyloctanoylcarnitine, L-1-pyrroline-3-hydroxy-5-carboxylate, dihydrocortisol, (6,8)-decadienoylcarnitine, (2E,4E,7E)-decatrienoylcarnitine, N-acetylaminooctanoic acid, dehydroepiandrosterone sulfate) were found to be associated with lung cancer [19]. The urinary smoking-related biomarkers of 259 lung cancer patients and 503 healthy controls showed that nicotine equivalents (TNE), 4-(methylnitrosamino)-1-3-(pyridyl)-1-butanol (NNAL), and cadmium (Cd) were higher in lung cancer patients. This suggests that smoking-related biomarkers may be an indicator for estimating lung cancer risk [20]. Urine samples from 46 lung cancer patients and 81 healthy controls were analyzed by gas chromatography-ion mobility spectrometer (GC-IMS) and electronic nose (e-nose), and the results showed that 8 VOCs (2-pentanone, 2-hexenal, 2-hexen-1-ol, hept-4-en-2-ol, 2-heptanone, 3-octen-2-one, 4-methylpentanol, and 4-methyl-octane) could identify lung cancer patients with Area Under the ROC Curve (AUC) of 0.91, sensitivity of 85%, specificity of 90%, and accuracy of 88% [21]. Headspace-solid phase microextraction gas chromatography coupled with mass spectrometry analysis of urine from 10 male stage IV lung cancer patients before and after chemotherapy revealed that 5 ketones (2-pentanone, 5-methyl-3-hexanone, 3-methyl-2-heptanone, 3-octanone, and 3-hepten-2-one), 5 cyclohexanes (gamma-terpinene, mesitylene, cyclohexyl isocyanate, isoterpinolene, and 3,4-dimethylstyrene), 4 alcohols (2-ethyl-1-hexanol, 1-octanol, butylatedhydroxytoluene, and thymol), one aldehyde (4-methylbenzaldehyde), 4 esters (ethylbenzoate, 3-hydroxy-2,4,4-trimethylpentyl-2-methylpropanoate, 2,2-dimethyl-1-(2-hydroxy-1-methylethyl) propyl 2-methylpropanoate and ethyl4-ethoxybenzoate) showed changes before and after chemotherapy. In particular, 2-pentanone showed a marked decrease in detection after chemotherapy [22]. Urine from 260 lung cancer patients was examined by headspace gas chromatography mass spectrometry, and it was found that urinary substances varied depending on the time period between the date of urine collection and the date of death. 12 VOCs (nonan-2-one, heptan-2-one, benzaldehyde, (Z)-oct-2-enoic acid, 5-ethyl-5-methyloxolan-2-one, propan-2-one, hexane-3,4-dione, 3,4-dimethylhexan-2-one, 2-methyl-5-methylsulfanylfuran, cyclohexanone, phenol, 4-methylpent-3-enoic acid, (E)-non-3-en-2-one and 1,2,4-triazole-3,4-diene-3,4-diamine) were elevated by death and 25 VOCs (3,4-dimethylthiophene, 1-(furan-2-yl)ethenone 4,7,7-trimethylbicyclo [4.1.0]hept-2-ene((+)-4-carene), 1-methyl-4propan-2-yl-7-oxabicyclo [2.2.1]heptane, 1-hydroperoxyhexane, 1-methyl-4-propan-2-ylbenzene(p-Cimene), 1,3,3-trimethyl-2-oxabicyclo [2.2.2]octane{Eucalyptol}, 1-methyl-4-propan-2-yicyclohexa-1.4-diene, 2,6-dimethyloct-7-en-2-ol, 5-(3,3-dimethyloxiran-2-yl)-3-methylpent-1-en-3-ol, 1-methyl-2-prop-1-en-2-ylbenzen, 3-methylpentan-3-ol, 2-(5-ethenyl-5-methyloxolan-2-yl)propan-2-ol, 2-methoxyphenol, 1,2,4,5-tetramethylbenezen, 5-methyl-2-propan-2-ylcyclohexan-1-ol (menthol), (1,4-dimethylpent-2-enyl) benzene, 2-methyl-5-prop-1-en-2-ylcyclohex-2-en-1-one (CARVONE), 1,1,4a-trimethyl-4,5,6,7-tetrahydro-3H-naphthalen-2-one, 2,6,6,10-tetramethyl-1-oxaspiro [4.5]dec-9-ene, 2-buta-1,3-dienyl-1,3,5-trimethylbenzene, (E)-1-(2,6,6-trimethylcyclohexa-1,3-dien-1-yl)but-2-en-1-one, and (5R)-5-methyl-2-propan-2-ylidenecyclohexan-1-one-(pulegone)) tended to decrease by the time of death [23].

### 2.2. VOCs of Bladder Cancer

Urine from 30 bladder cancer patients and 30 healthy controls were analyzed by multicapillary column ion mobility spectrometry (MCC/IMS). 82 peaks were detected, of which 8 peaks showed significant differences. A six-step decision tree was created with benzylaldehyde or benzofuran, ammonia, toluene, hexylbenzene, cyclohexene 1-methyl-4-(1-methylethylidene), and acetylvaleryl showed a sensitivity of 90% and specificity of 100% [24]. A reanalysis of 422 studies, which analyzed urinary bladder cancer markers in traditional tobacco users and electronic cigarettes (e-cigarettes) users, showed that urinary bladder cancer markers were elevated in traditional tobacco users but not in e-cigarettes users. Urinary bladder cancer markers (1-hydroxynaphthalene, 1-hydroxyphenanthrene, 1-hydroxypyrene, 2-hydroxyfluorene, 2-hydroxynaphthalene, 3-hydroxyfluorene, 2-carbamoylethylmercapturic acid, 4-hydroxy-2-buten-1-yl mercapturic acid, 4-(methylnitrosamino)-1-(3 pyridyl)-1-butanone, N’-nitrosonornicotine (NNN)) were identified [25]. VOCs were extracted from the urine of 40 patients with bladder cancer and 57 healthy controls by head space solid phase microextraction (SPME) and analyzed by gas chromatography time of flight mass spectrometry (GC GC TOF MS) and 12 VOCs (pyridine, 4-propylbenzaldehyde, 2,3-heptanedione, heptanal, 4-methyl-3-pentenoicacid, 1-heptanol, 1-octen-3-one, 2,6-dimethyaniline, 11-hexadecen-1-ol, 4,6-dimethyl-2-heptanone, 2-butyl-1-octanol, 2,3,5-trimethylfuran) were detected only in patients with bladder cancer. In addition, butyrolactone, 2-methoxyphenol, 3-methoxy-5-methylphenol, 1-(2,6,6-trimethylcyclohexa-1,3-dien-1-yl)-2-buten 1-one, nootkatone and 1-(2,6,6-trimethyl-1-cyclohexenyl)-2-buten-1-one were identified as biomarker candidates [26]. Urine from 75 recurrent bladder cancer patients and 84 recurrence-free patients was analyzed by headspace sampling utilized solid-phase microextraction and gas chromatography-mass spectrometry analysis showed that 10 VOC markers (nonanal, 2-ethylhexan-1-ol, 1,1,4a-trimethyl-4,5,6,7-tetrahydro-3H-naphthalen-2-one, 5-ethyl-3-methyloxolan-2-one, phenol, 4-methylpent-3-enoicacid, 2-methoxyphenol, 3-methylheptan-2-one, 1,2,4,5-tetramethylbenzene and heptan-2-one) were detected. The diagnostic model was constructed with six of them (nonanal, 2-ethylhexan-1-ol, 5-ethyl-3-methyloxolan-2-one, heptan-2-one, 1,1,4a-trimethyl-4,5,6,7-tetrahydro-3H-naphthalen-2-one and propan-2-one) and showed AUC of 0.80, sensitivity of 0.71 and specificity of 0.80 [27].

### 2.3. VOCs of Prostate Cancer

Urine from 247 prostate cancer patients and 139 non-prostate cancer patients (biopsy negative) were analyzed by gas chromatography and mass spectrometry (GC-MS) and 22,538 VOCs were detected. A diagnostic model was constructed and tested using machine learning tools based on the obtained data, and the AUC was 0.88. In the urine of prostate cancer patients, acetophenone, dodecamethylcyclohexasiloxane, galaxolide, versalide, decamethylcyclopentasiloxane, tridecane, 1,1,3,3,5,5,7,7-octamethyl-7-(2-methylpropoxy)tetrasiloxan-1-ol, octanal, 2-(phenylmethylene)-, decanal, and 1-decene were detected with the largest significant differences. methyl dihydrojasmonate, L-phenylephrine, 2-phenyl-3-decyn-1-ol, N,N-diisopropylethylamine were significantly detected in the low grade, while methyl glyphosate, semicarbazide, 5-ethyl-1,2,3,4-tetrahydronaphthalene, norcodeine, N-trimethylsilyl-, trimethylsilyl ether, 2,5-di-tert-butyl-1,4-benzoquinone and butane-1,4-diol were significantly detected in the high grade [28]. Urinary substances from 66 prostate cancer patients and 87 healthy controls were analyzed by gas chromatography-ion mobility spectrometry (GC-IMS) and 86 substance peak heights were detected. Two diagnostic models were constructed using 4 VOCs: up-regulated furan-3-methanol, down-regulated (E, E)-octadeca-2,4-dienal, 2-ethylhexan-1-ol and 2-undecen-1-al, and the AUC was 0.955 and 0.981, respectively [29]. Urine from 26 Prostate cancer patients and 30 healthy controls was analyzed by headspace solid phase microextraction combined with gas chromatography-mass spectrometry (HS-SPME/GC-MS) detected 147 endogenous volatile organic metabolites (VOMs) and the 13 most important peaks (dimethyl disulfide, 4-heptanone, o-cymene, p-cymenene, dihydromyrcenol, 2-ethyl-1-hexanol, menthol, D-carvone, β-damascenone, phenol, 4-methyphenol, β-ionone; 2-bromophenol) for cancer patient were identified [30].

### 2.4. VOCs of Breast Cancer

Urine from 80 breast cancer patients and 88 healthy controls was analyzed by headspace-solid phase microextraction (HS-SPME) combined with gas chromatography-high resolution mass spectrometry (GC-HRMS), 134 VOCs were detected. Among them, 8 VOCs ((E)-piperitol, 4′-hydroxyacetophenone, 4-nitrophenyl butyrate, 2,4-dihydroxybenzaldehyde, 3-acetyl-2,5-dimethyl furan, 2-chloro-4-(2,3-dichlorophenyl) benzoic acid, 2,4-dimethylbenzaldehyde, pentandioic acid and (p-t-butylphenyl) ester) were used to construct diagnostic model which showed sensitivity of 76% and specificity of 92.3% [31]. Urine samples from breast cancer patients were analyzed by gas chromatography-mass spectrometry (GC-MS), and an artificial intelligence-based classification algorithm was constructed. The classification rate was 92.31%, sensitivity was 100%, and specificity was 85.71%. The classification rate was 75%, sensitivity was 100%, and specificity was 50% when the classification algorithm was constructed using eNose data [32].

### 2.5. VOCs of Other Cancers

To investigate urinary VOCs of clear cell renal cell carcinoma (ccRCC), a type of renal cancer, the urine of 233 pretreatment ccRCC patients and 43 healthy controls was analyzed by stir-bar sorptive extraction coupled with thermal desorption gas chromatography/mass spectrometry (SBSE-TD-GC-MS) and 6218 potential VOCs were detected. Among them, 56 VOC markers with a *p*-value < 0.05 were identified in ccRCC patients and 227 VOC markers in healthy controls. Of these, 10 VOC markers (1-hexanol,2-ethyl-, heptadecanolid, cis-vaccenic acid, decane,3-methyl-, naphthalene, 1,2,3,5,6,7,8, 8a-octahydro-1, 8a-dimethyl-7-(1-methylethenyl)-, [1R-(1.alpha.,7.beta.,8a.alpha.)]-, 1,4-bis (trimethylsilyl) benzene, carbonicacid, decyl nonyl ester, l-(+)-ascorbicacid 2,6-dihexadecanoate, cyclohexene, 6-ethenyl-6-methyl-1 (1-methylethyl)-3-(1 methylethylidene)-,(S)- and 2-methyl-6-(p-tolyl)hept-2-en-4-ol) were elevated in ccRCC patients. The diagnostic model was constructed and AUC was 0.94, sensitivity was 86%, and specificity was 92% [33]. Urine samples from 38 esophageal cancer patients and 32 healthy controls were analyzed by gas chromatography ion mobility spectrometry (GC-IMS), and 8 VOCs (cyclohexanone-D, 2,3-butandiol, methyl decanoate, (E)-ethyl-2-hexenoate, 2-acetylfuran, 2-methyl-butanoic acid methyl ester, 2-isopropyl-3-methoxy pyrazine, dimethyl trisulfide) were used to construct the diagnostic model which showed the AUC of 0.874, the sensitivity of 84.2%, and the specificity of 90.6% [34]. Blood and urine samples from 101 kidney cancer patients, 26 lung cancer patients, 26 gastric cancer patients, and 152 healthy controls were analyzed by gas chromatography-mass spectrometry (GC-MS) and more than 100 VOCs were detected. Of these, it turned out that in the construction of blood diagnostic model, pentanal, dihydrofuran, pentyl furan, hexanal, dodecane, hexane, and methyl butanal were important, in the construction of urine diagnostic model, 2-heptanone, decane, thanone, pentanone, octane and dodecane were important, and in the construction of blood and urine diagnostic model, hexanal, rentanal, dihydrofuran, dodecane, hexane, pentyl furan, methyl butanal were important. The AUC of the blood model was 0.91, sensitivity was 0.90, specificity was 0.89, and accuracy was 0.92. The AUC of the urine model was 0.83, sensitivity was 0.78, specificity was 0.70, and accuracy was 0.82. The AUC, sensitivity, specificity, and accuracy of the combined model of blood and urine were 0.93, 0.92, 0.91, and 0.94, respectively [35]. The urine of 85 cancer patients (42 patients with lung cancer, 25—colon, 3—stomach, 2—prostate, 2—esophageal, 2—pancreas, 2—kidney, 1—ovarian, 1—cervical, 1—skin, 1—liver) and 89 healthy controls were analyzed for VOCs by gas chromatography with mass spectrometry (GC-MS), and it was found that 2-pentanone, acetonitrile and benzene were elevated in cancer patients, while 2-butanone showed a decreasing trend. Fourteen pairs (benzene/acetone, acetonitrile/acetone, 2-butanone/acetone, acetonitrile/dimethyl sulfide, toluene/dimethyl sulfide, acetone/dimethyl sulfide, benzene/dimethyl disulfide, acetonitrile/dimethyl disulfide, dimethylsulfide/dimethyl disulfide, 2-butanone/dimethyl disulfide, 2-pentanone/dimethyl disulfide, butanal/dimethyl disulfide, acetonitrile/dimethyl trisulfide, 2-pentanone/dimethyl trisulfide) were used to construct diagnostic model which showed the ROC of 0.886, sensitivity of 91% and specificity of 82% [36]. As described above, the identification of VOCs in urine of cancer patients by mass spectrometry and the construction of a diagnostic model using the analysis data are underway.

## 3. Detection of Cancer Odors by Organisms

Olfactory receptors detect a variety of odors. It has been reported that there are organisms that can detect the unique smells of cancer (Table 2 and Table 3). It is expected that this ability will be applied to cancer diagnosis, and some of them have been implemented in society.

### 3.1. Cancer Diagnosis by Dogs

When dogs were trained to learn the odor of the urine of 27 bladder cancer patients and that of non-bladder cancer controls to identify the urine of bladder cancer patients, it was observed that in some cases the dogs were able to respond to the urine of bladder cancer patients. This suggests that the urine of bladder cancer patients contains odorants derived from bladder cancer [37]. Prostate cancer is confirmed by prostate biopsy, and the dog’s sense of smell has attracted attention as a non-invasive method. Seven dogs were tested to discriminate between 78 samples of urine from patients with prostate cancer and 73 samples of urine from controls, five dogs had a sensitivity greater than 73% and six dogs had a specificity greater than 75% [38]. When dogs were tested on 93 lung cancer patients with exhaled breath and urine, sensitivity rates ranged from 56 to 76% and specificity rates ranged from 8.3 to 33.3% [39]. When dogs were tested to identify colorectal cancer using exhaled breath and watery stool samples from colorectal cancer (CRC) patients, sensitivity was 97% and specificity was 99%. The results suggest that cancer-specific chemical compounds are circulating in the body [40]. Dogs were tested to see if they could discriminate between the blood of 42 ovarian cancer patients and that of 210 healthy subjects and the sensitivity was 97%. 29 of the 42 patients died within 5 years [41]. To determine whether dogs can be used to diagnose ovarian carcinoma, ovarian cancer tissues and blood from patients were used in the tests and found that in tissue tests, sensitivity was 100% and specificity was 95%, and in blood tests, sensitivity was 100% and specificity was 98%. This suggests that substances in ovarian cancer tissues are circulating in the blood [42]. When dogs were asked to identify the odor of ovarian cancer tissues, they showed 100% sensitivity and 97.5% specificity [43].

**Table 2:** Cancer identification by dogs.

Cancer Type	Sample	Sensitivity (%)	Specificity (%)	References
Prostate	Urine	73	75	[38]
Lung	Exhaled breath and urine	56–76	8.3–33.3	[39]
Colorectal	Exhaled breath and watery stool	97	99	[40]
Ovarian	Blood	97	99	[41]
Ovarian	Blood	100	98	[42]
Ovarian	Tissue	100	97.5	[43]

**Table 3:** Identification of urine from cancer patients by nematodes.

Cancer Type	AUC	Sensitivity (%)	Specificity (%)	References
Pancreatic		84.6	60	[44]
Esophageal, Gastric, Colorectal, Gallbladder, Cholangiocarcinoma, Breast, Malignant Lymphoma, and Acute myeloid leukemia	0.9032	87.5	90.2	[45]
Prostate		95	85	[46]
Colorectal	0.716			[47]
Gastrointestinal	0.860	>80	88.2	[48]

### 3.2. Cancer Diagnosis Using Nematodes

The Nematode-NOSE (N-NOSE) system, a system in which nematodes identify urine derived from cancer patients, was constructed to detect pancreatic cancer with nematodes and tested in the urine of 104 pancreatic cancer patients and 95 healthy controls, and the sensitivity was 84.6% and specificity was 60%. The N-NOSE was also able to detect stage 0 and stage I pancreatic cancer [44]. Urine from 32 cancer patients (esophageal, gastric, colorectal, gallbladder, cholangiocarcinoma, breast, malignant lymphoma, and acute myeloid leukemia) was diluted and tested by N-NOSE. The AUC for the 10-fold dilution was 0.9188, and the AUC for the 100-fold dilution was 0.9032. Combining these data to make a diagnosis resulted in a sensitivity of 87.5%. It was also effective in diagnosing early-stage cancer, which is difficult to do with the blood cancer markers CEA, CA19-9 and CA15-3 [45]. To determine whether *C. elegans* can identify prostate cancer in urine, we tested the response to 2-octonone and pentanal, VOC candidates for prostate cancer. The nematodes responded at concentrations of 64 mM and 640 mM for 2-octonone, but only at the higher concentration condition of 940 mM for pentanal. In addition, diagnostic testing of urine samples from prostate cancer patients at 1:100 diluted urine yielded an accuracy of 81% [46]. Urine samples from 46 colorectal cancer patients and 32 gastric cancer patients were tested with N-NOSE and compared to the existing blood cancer markers CEA and CA19-9 for diagnosis. The AUCs for CEA and CA19-9 were 0.688 and 0.597, respectively, while the AUC for N-NOSE was 0.716. This indicates that N-NOSE is more accurate than existing molecular markers for diagnosis [47]. When N-NOSE was tested in the urine of 180 gastrointestinal cancer patients and 76 healthy controls, the AUC exceeded 0.8, exceeding the results of diagnostic tests using the existing blood markers CEA and CA19-9 [48]. When nematodes were tested in the urine of pancreas-specific KrasG12D mice, a mouse model of pancreatic cancer, they were found to be attracted to the urine of the pancreatic cancer model mice, whereas they were not attracted to the urine of healthy mice. This mouse model may help elucidate the mechanism of pancreatic cancer detection by nematodes [49]. To determine whether nematodes can diagnose canine cancer, the urine of dogs with cancer was tested and found that sensitivity was 85% and specificity was 90% [50]. It was reported that N-NOSE can distinguish 15 types of human cancer (stomach, colon-rectum, lung, breast, pancreas, liver, prostate, uterus, esophagus, gallbladder, bile duct, kidney, urinary bladder, ovary, and oropharynx cancers). The application of N-NOSE to cancer detection in dogs and cats was examined and it was found to be significantly discriminating [51].

### 3.3. Cancer Diagnosis by Other Organisms

Lung cancer cells (LKR and LLC) were transplanted into mice and it was observed that the mice could identify cancer-derived substances using urine of the mouse models. Analysis of the chemicals in the urine showed differences in the amounts of some substances between cancer and control mice, with cancer mice showing elevated levels of 2-ethyl hexanoic acid and decreased levels of 5-hepten-2-one [52]. In lung cancer, exhaled air contains toluene. When rats were exposed to air containing toluene (toluene-spiked breath samples), their behavior was observed, and the rats learned to smell toluene, they were able to identify toluene-spiked breath and their memories remained 45 days later [53]. When the rats were stimulated with urine from patient-derived xenograft mice with triple negative breast cancer to learn the odor of cancer, they spent 20% more time at the patient-derived xenograft (PDX) urine spot. The urinary chemicals was examined and found to be altered in PDX compared to healthy mice [54]. A brain-based sensing technology using the odor of culture media from non-small cell lung cancer (NSCLC) and small cell lung cancer (SCLC) cell lines was developed to measure the response of honeybee brain olfactory, distinguishing human NSCLC and SCLC cell lines from ’synthetic healthy’ human breath by measuring the response of honeybee brain olfactory neurons to the odor [55].

## 4. *C. elegans* Chemotaxis

*C. elegans* has olfactory receptors to detect cancer markers [56]. *Cat*-2 mutant nematodes with low dopamine (DA) synthesis showed predominantly decreased chemotaxis index (CI). When the mutant *C. elegans* were trained in the presence of exogenous DA (10 mM), an increase in CI value was observed. This suggests that *dop-1* and *dop-3* DA receptors are involved in memory retention [57]. Aberrant chemotaxis to odorants such as diacetyl and isoamyl alcohol was observed in *egl-4* mutant *C. elegans* encoding a cGMP-dependent protein kinase (PKG). The AIY interneuron of the *egl-4* mutant functions to repress chemotaxis. This response was seen only in signals from two upstream olfactory neurons, anterior worm amphid neuron A (AWA) and anterior worm amphid neuron C (AWC). This suggests that sensory signals are inappropriately accumulated in the *egl-4* mutant. Furthermore, normal *egl-4* expression in the *egl-4* mutant restored chemotaxis, suggesting that EGL-4/PKG suppresses the abnormal accumulation of signals from olfactory neurons and converts olfactory stimuli to chemotactic behavior [58]. Exp-1, an excitatory GABA receptor, regulates DAF-7/TGF-β levels. When *exp-1* was mutated, the chemotaxis index to isoamyl alcohol, benzaldehyde, and 2-butanone was decreased. AWC senses attractive odors and ASI senses food-dependent odors. The secreted DAF-7 or alternate TGF-β pathway, which is regulated by EXP-1 and STR-2, is important for neuronal connections between the two [59]. The *prk-1* mutant, a PIM-related kinase, showed decreased chemotaxis to butanone and decreased avoidance to 1-octanol [60]. DEET (N, N-diethyl-meta-toluamide) is used as an ingredient in insect repellents, and in the presence of DEET, the chemotaxis of nematodes to isoamyl alcohol, butanone and diacetyl is decreased [61]. Although isoamyl alcohol is an attractive odor of *C. elegans*, stimulation with isoamyl alcohol increases longevity in a FoxO homolog DAF-16-dependent manner. Although acetic acid is a repellent component, stimulation with acetic acid promotes thermotolerance in a DAF-16-independent manner [62]. Chemotaxis to 2-heptanone is decreased in *odr-1* or *str-2* mutants [63]. Ras GTPase activating proteins (RasGAPs) are important for learning and memory, and inhibition of the RasGAPs *gap-1*, *gap-2*, and *gap-3* reduced chemotaxis [64]. *C. elegans* is attracted to lactic acid bacteria cultured on citrate media, and this chemotaxis is mediated by the diacetyl odor receptor, ODR-10. Furthermore, nematodes were found to be attracted to diacetyl produced by lactic acid bacteria [65]. RNA interference (RNAi) screening to identify the receptors required for response to odorants revealed 194 olfactory receptor genes associated with 11 odorants (attractants: isoamyl alcohol, benzaldehyde, butanone, pentanedione, pyrazine, trimethylthiazole, repellents: nonanone, octanol, high isoamyl alcohol, high benzaldehyde, high diacetyl). Multiple receptors were involved in the response to Odorants other than high isoamyl alcohol, and about 50 receptors were involved in the response to benzaldehyde and pyrazine. It is found that SRI-14 is required for sensing high concentrations of diacetyl, and functions in ASH neurons [66]. The chemotaxis index of *C. elegans* to explosive substances and their precursors was examined and found to be 0.83 for nitroglycerine, 0.79 for acetone and 2-butanone, 0.50 for nitromethane, 0.47 for nitroglycerine and hexamine, and 0.46 for cyclohexanone. The *odr-1*, *odr-7*, and AWC olfactory neurons marker *ceh-36* were found to be important for cyclohexanone chemotaxis [67]. Attraction to benzaldehyde is no longer observed in the odr-3 mutant [68]. *Odr-10* encodes a 7-transmembrane receptor that detects odorant diacetyl and the chemotaxis to diacetyl was defective in the *odr-10* mutant. *Odr-10* is expressed in the AWA sensory cilia and its expression is regulated by the transcription factor *odr-7* [69]. Thus, various olfactory receptor-related genes are involved in chemotaxis in *C. elegans*.

## 5. Detection of Cancer Markers by Artificial Sensors

The development of sensors that react to VOCs is also underway. An organic compounds detector consisting of a titanium-based inorganic material and peptides was fabricated and found to discriminate 15 odor molecules classified as alcohols, ketones, aldehydes, esters, and acids. Diagnostic model was constructed using measurement data of odor molecules in the breath of healthy populations, patients of lung cancer, upper digestive tract cancer, and lower digestive tract cancer by the detector, showing identification of each group with an accuracy of more than 90% [70]. The commercially available carbon polymer array electronic nose (Cyranose C320, Sensigent, Baldwin Park, CA, USA) was tested on urine from 133 patients with prostate cancer and 139 healthy controls. The test correctly identified 110 out of 133 patients with prostate cancer and 123 out of 139 healthy controls [71]. TC-Sniffer, which is developed to diagnose urinary bladder cancer (UBC), achieved an accuracy of 92.95% in only five UBC training samples detecting VOCs in urine. Furthermore, it was able to distinguish between nonmuscle-invasive bladder cancer (NMIBC) and muscle-invasive bladder cancer (MIBC) [72]. The electronic nose was used to detect breath-derived VOCs before and after chemoradiotherapy in 45 patients with rectal cancer, and diagnostic model was constructed by machine-learning, showing the AUC of 0.63 [73]. Diagnostic testing of chondrosarcoma by electronic nose in 25 healthy controls, 24 patients with chondrosarcoma and 8 benign lesions showed a sensitivity of 75%, specificity of 65%, and AUC of 0.66 [74]. Graphene was found to adsorb ethanol, benzene and toluene and was shown to be a sensor for these compounds [75]. A hybridized porous zeolitic imidazolate (ZIF-8) based metal–organic frameworks (MOFs) and laser-scribed graphene (LSG) structure chemoresistive sensor was developed to detect acetone, ethanol, methanol and formaldehyde in exhaled breath. A diagnostic model was constructed by machine learning from data obtained from lung cancer patients, and the accuracy was 96.5% [76]. Metal oxide biosensor detection of urinary VOCs in patients with genitourinary cancer revealed that methane, iso-butane, hydrogen, and ethanol were diagnostic [77]. Ti_3_C_2_TXenes can be used as sensors for benzene, cyclohexane, isoprene, cyclopropanone, propanol, and butyraldehyde butanal, and thus can be applied to the diagnosis of lung cancer [78]. A polyvinylidene fluoride (PVDF)-based colorimetric sensor array has been developed, which can detect 28 kinds of disease-related VOCs (toluene, acetic anhydride, propionic acid, benzene, methacrolein, formic acid, acetonitrile, n-pentane, butyric acid, valeraldehyde, formaldehyde, acetaldehyde, 1-propanol, n-butyl acetate, acetone, n-hexadecane, 4-methyloctane, butyraldehyde, hexane, butylamine, 2,5-dimethylfuran, heptane, 1,2,4-trimethylbenzene, benzyl alcohol, nonanal, methyl phenylacetate, hexylcyclohexane, 2-methyl-1,3-butadiene) [79]. A gas sensor based on transition metal (Fe, Ni, and Pd) doped olympicene was developed and formaldehyde could be detected [80]. GC-MS analysis of breath samples of hepatocellular cancer patients revealed phenol 2,2 methylene bis [6-(1,1-dimethyl ethyl)-4-methyl] (MBMBP) as a marker. gold nanoparticles-based sensor was developed and found to be capable of detecting MBMBP [81]. Development of such biosensors is underway.

## 6. Conclusions and Future Directions

As mentioned above, the use of urine for noninvasive cancer diagnosis is being attempted. Urine contains a variety of VOCs, and it has been found that VOCs specific to cancer patients are present. Detection methods in cancer diagnosis are being studied to improve accuracy in the fields of detection by mass spectrometry, organisms, and biosensors. The greatest advantage of mass spectrometer detection is that it can detect a large number of substances. Therefore, it is important to construct better diagnostic models from the obtained data. Although many diagnostic models have been constructed so far, the creation of truly reliable models is an issue for the future. It will also be necessary in clinical practice to be able to process large amounts of data in a short period of time. The major advantage of biological detection is that the receptor can comprehensively sense and respond to substances in urine. Thus, it may have the ability to answer the question of cancer or not from a large number of input factors. However, it is difficult to obtain objective data on what substances in the urine responded to the diagnostic result. In the future, it will be necessary to elucidate the mechanisms involved. In particular, cancer diagnosis using nematodes has already been implemented in society and is making a significant contribution to early cancer detection. As for the phenomenon that the smell of cancer can be identified by the olfactory receptors of organisms, the detailed mechanism of what kind of substances react with what kind of receptors will need to be elucidated in the future. The advantage of biosensor-based detection is that diagnosis can be made on exhaled breath. While the ability to detect specific VOCs with a simple diagnosis is a unique feature, further evolution may be needed in terms of making a comprehensive judgment from a large number of VOCs. In the future, it is necessary to improve the accuracy of each diagnostic method to the point that the exact cancer type can be determined. It is hoped that the discovery of novel biomarkers and their diagnostic methods will make the early detection of cancer increasingly easier.

## Figures and Tables

**Table 1:** Urinary chemicals of cancer patients.

Detcted Chemicals	Cancer Type	References
N-acetyl-S-methyl-L-cysteine, 3/4-methylhippuric acid, 2-methyl hippuric acid, N-acetyl-S-(N-methyl carbamoyl)-L-cysteine, N-acetyl-S-(2-carbamoylethyl)-L-cysteine, N-acetyl-S-(2-carboxyethyl)-L-cysteine, N-acetyl-S-(3-hydrox ypropyl-1-methyl)-L-cysteine, N-acetyl-S-(3-carboxy-2-propyl)-L cysteine, N-acetyl-S-(2-carbamoylethyl)-L-cysteine and N-acetyl-S (3,4-dihydroxybutyl)-L-cysteine	Lung	[19]
nicotine equivalents (TNE),4-(methylnitrosamino)-1-3-(pyridyl)-1-butanol (NNAL) and cadmium (Cd)	Lung	[20]
2-pentanone, 2-hexenal, 2-hexen-1-ol, hept-4-en-2-ol, 2-heptanone, 3-octen-2-one, 4-methylpentanol and 4-methyl-octane	Lung	[21]
2-pentanone, 5-methyl-3-hexanone, 3-methyl-2-heptanone, 3-octanone, 3-hepten-2-one gamma-terpinene, mesitylene, cyclohexyl isocyanate, isoterpinolene, 3,4-dimethylstyrene 2-ethyl-1-hexanol, 1-octanol, butylatedhydroxytoluene and thymol 4-methylbenzaldehyde, ethylbenzoate, 3-hydroxy-2,4,4-trimethylpentyl-2-methylpropanoate, 2,2-dimethyl-1-(2-hydroxy-1-methylethyl)propyl 2-methylpropanoate, ethyl4-ethoxybenzoate	Lung	[22]
onan-2-one, heptan-2-one, benzaldehyde, (Z)-oct-2-enoic acid, 5-ethyl-5-methyloxolan-2-one, propan-2-one, hexane-3,4-dione, 3,4-dimethylhexan-2-one, 2-methyl-5-methylsulfanylfuran, cyclohexanone, phenol, 4-methylpent-3-enoic acid, (E)-non-3-en-2-one and 1,2,4-triazole-3,4-diene-3,4-diamine	Lung	[23]
benzylaldehyde, benzofurane, ammonia, toluene, hexylbenzene, cyclohexene 1-methyl-4-(1-methylethylidene) and acetyl valeryl	Bladder	[24]
1-hydroxynaphthalene (1-NAP), 1-hydroxyphenanthrene (1-PHE), 1-hydroxypyrene (1-PYR), 2-hydroxyfluorene (2-FLU), 2-hydroxynaphthalene (2-NAP), 3-hydroxyfluorene (3-FLU), 2-carbamoylethylmercapturic acid (AAMA), 4-hydroxy-2-buten-1-yl-mercapturic acid (MHBMA), 4-(methylnitrosamino)-1-(3 pyridyl)-1-butanone (NNAL) and N’-nitrosonornicotine (NNN)	Bladder	[25]
pyridine, 4-propylbenzaldehyde, 2,3-heptanedione, heptanal, 4-methyl-3-pentenoicacid, 1-heptanol, 1-octen-3-one, 2,6-dimethyaniline, 11-hexadecen-1-ol, 4,6-dimethyl-2-heptanone, 2-butyl-1-octanol, 2,3,5-trimethylfuran, butyrolactone, 2-methoxyphenol, 3-methoxy-5-methylphenol, 1-(2,6,6-trimethylcyclohexa-1,3-dien-1-yl)-2-buten 1-one, nootkatone and 1-(2,6,6-trimethyl-1-cyclohexenyl)-2-buten-1-one	Bladder	[26]
nonanal, 2-ethylhexan-1-ol, 1,1,4a-trimethyl-4,5,6,7-tetrahydro-3H naphthalen-2-one, 5-ethyl-3-methyloxolan-2-one, phenol, 4-methylpent-3-enoicacid, 2-methoxyphenol, 3-methylheptan-2-one, 1,2,4,5-tetramethylbenzene and heptan-2-one	Bladder	[27]
acetophenone, dodecamethylcyclohexasiloxane, galaxolide, versalide, decamethylcyclopentasiloxane, tridecane,1,1,3,3,5,5,7,7-Octamethyl-7-(2-methylpropoxy)tetrasiloxan-1-ol, octanal, 2-(phenylmethylene)-, decanal and 1-decene	Prostate	[28]
furan-3-methanol, (E, E)-octadeca-2,4-dienal, 2-ethylhexan-1-ol and 2-undecen-1-al	Prostate	[29]
dimethyl disulfide, 4-heptanone, o-cymene, p-cymenene, dihydromyrcenol, 2-ethyl-1-hexanol, menthol, D-carvone, β-damascenone, phenol, 4-methyphenol, β-ionone and 2-bromophenol	Prostate	[30]
(E)-piperitol,4′-hydroxyacetophenone, 4-nitrophenyl butyrate, 2,4-dihydroxybenzaldehyde, 3-acetyl-2,5-dimethyl furan, 2-chloro-4-(2,3-dichlorophenyl) benzoic acid, 2,4-dimethylbenzaldehyde, pentandioic acid and (p-t-butylphenyl) ester	Breast	[31]
8-oxo-7,8-dihydro-2’-deoxyguanosine	Breast	[32]
1-hexanol,2-ethyl-, heptadecanolid, cis-vaccenic acid, decane,3-methyl-, naphthalene, 1,2,3,5,6,7,8,8a-octahydro-1,8a dimethyl-7-(1-methylethenyl)-, [1R-(1.alpha.,7.beta.,8a.alpha.)]-, 1,4-bis(trimethylsilyl)benzene, carbonicacid, decyl nonyl ester, l-(+)-ascorbicacid 2,6-dihexadecanoate, cyclohexene, 6-ethenyl-6-methyl-1 (1-methylethyl)-3-(1 methylethylidene)-,(S)- and 2-methyl-6-(p-tolyl)hept-2-en-4-ol	Renal	[33]
cyclohexanone-D, 2,3-butandiol, methyl decanoate, (E)-ethyl-2-hexenoate, 2-acetylfuran, 2-methyl-butanoic acid methyl ester, 2-isopropyl-3-methoxy pyrazine and dimethyl trisulfide	Esophageal	[34]
2-heptanone, decane, thanone, pentanone, octane and dodecane	Kidney, Lung and Gastric	[35]
2-pentanone, acetonitrile and benzene	Lung, Colon, Stomach, Prostate, Esophageal, Pancreas, Kidney, Ovarian, Cervical, Skin and Liver	[36]
